# High resolution temporal profiles in the Emissions Database for Global Atmospheric Research

**DOI:** 10.1038/s41597-020-0462-2

**Published:** 2020-04-17

**Authors:** Monica Crippa, Efisio Solazzo, Ganlin Huang, Diego Guizzardi, Ernest Koffi, Marilena Muntean, Christian Schieberle, Rainer Friedrich, Greet Janssens-Maenhout

**Affiliations:** 1 European Commission, Joint Research Centre (JRC), Ispra, Italy; 2 Institute of Energy Economics and Rational Energy Use (IER), Universität Stuttgart, Hessbruehlstr. 49a, 70565 Stuttgart, Germany

**Keywords:** Atmospheric science, Environmental impact

## Abstract

Emissions into the atmosphere from human activities show marked temporal variations, from inter-annual to hourly levels. The consolidated practice of calculating yearly emissions follows the same temporal allocation of the underlying annual statistics. However, yearly emissions might not reflect heavy pollution episodes, seasonal trends, or any time-dependant atmospheric process. This study develops high-time resolution profiles for air pollutants and greenhouse gases co- emitted by anthropogenic sources in support of atmospheric modelling, Earth observation communities and decision makers. The key novelties of the Emissions Database for Global Atmospheric Research (EDGAR) temporal profiles are the development of (i) country/region- and sector- specific yearly profiles for all sources, (ii) time dependent yearly profiles for sources with inter-annual variability of their seasonal pattern, (iii) country- specific weekly and daily profiles to represent hourly emissions, (iv) a flexible system to compute hourly emissions including input from different users. This work creates a harmonized emission temporal distribution to be applied to any emission database as input for atmospheric models, thus promoting homogeneity in inter-comparison exercises.

## Abstract (summary)

**Table:** 

Measurement(s)	air pollution • greenhouse gas • temporal measurement
Technology Type(s)	computational modeling technique
Factor Type(s)	sector • geographic location
Sample Characteristic - Environment	climate system
Sample Characteristic - Location	Earth (planet)

Machine-accessible metadata file describing the reported data: 10.6084/m9.figshare.12052887

## Background & summary

Owing to the increasing need of integrated climate and air quality policies, implementation of mitigation and adaptation measures and the application of top-down approaches using Earth observations to address environmental issues, real-time mapping of human emissions of greenhouse gases (GHGs) and air pollutants is becoming of high relevance^1–3^. In this respect, annual emission estimates might be unable to reflect acute heavy pollution episodes^4^, and to model the dynamics of atmospheric formation of pollution loadings during different periods of the year and different hours of the day^5^. Even moderate total annual emissions for a certain region can be affected by periodic intensive emissions. Temporally disaggregated emissions are also essential to estimate surface emission fluxes of atmospheric composition (including reactive gases and greenhouse gases), and are a required input for advanced chemical transport models (CTMs)^6,7^, which simulate hourly concentrations of air pollutants^8–10^ and are used in support of the legislation.

Human activities emit greenhouse gases and air pollutants with different temporal variation, depending on the type of activity. A review of temporal profiles from literature and profiles used by atmospheric models is conducted, despite the relatively limited amount of studies in this field. Few studies focus on the monthly variations of the emissions^4,11,12^, while little attention has been paid to daily and hourly variations. In addition, the spatial and sectorial resolutions of relevant works are also limited, often covering only a specific region or sector^8,13,14^. Until recently, global emission inventories mainly provide anthropogenic emissions on annual or monthly time series^15–19^.

In this study, sector- and country-specific temporal profiles (EDGAR_temporal_profiles_r1, available at figshare)^20^ are developed and integrated into the EDGAR database to produce monthly and hourly emission time series and gridmaps. The novelty of this work relies on the development of (i) country/region- and sector- specific yearly splitting profiles for all EDGAR emissions, (ii) time dependent yearly profiles for emission sectors with inter-annual variability of their seasonal patterns, (iii) country-specific weekly and daily profiles to represent the hourly variations of the emissions, taking into account country specific holidays and weekends definition, (iv) a flexible system to compute hourly emissions including input from different users. The EDGAR temporal profiles can be applied to any air pollutant (SO_2_, NOx, CO, NMVOC, NH_3_, PM_10_, PM_2.5_, BC, OC) and GHG (CO_2_, CH_4_, N_2_O) since they have been developed for all anthropogenic emissions sources which often co-emit a variety of pollutants. As an exemplification, in this work we also compute monthly emission time series of EDGARv4.3.2 for all pollutants (CO_2_, CH_4_, N_2_O, SO_2_, NOx, CO, NMVOC, NH_3_, PM_10_, PM_2.5_, BC, OC). The EDGAR temporal profiles system is easily updated when new information is available and can be applied to any global and regional database.

As applications to modelling and policy related fields, we would like to point out that:

- EDGAR is used as default emission inventory in air quality modelling (e.g. Hemispheric Transport of Air Pollution (HTAP_v1 and HTAP_v2)^21^, FP7 PEGASOS^15^, etc.) and model intercomparisons (e.g. HTAP, AQMEII, EURODELTA, etc.^7^). In particular, global and regional models employ their own emission time distribution, giving rise to heterogeneity of results and difficult interpretation of model differences. Using a common emission temporal distribution could promote homogeneity in intercomparison exercises.
- EDGAR emission grids are used by the Global Carbon Project as a-priori fluxes to run inverse atmospheric modeling^22^. With the increased interest in the monitoring and verification of GHGs using top-down measurements temporal profiles are equally needed.
- EDGAR can support analysis in the agricultural sector providing more accurate information to assess impacts on crops^23^.
- EDGAR is widely used in support of policy design, treaty compliances, Intergovernamental Panel on Climate Change (IPCC) and emission verification (http://verify.lsce.ipsl.fr/). High time resolution emissions enhance the monitoring capability of EDGAR by adding seasonal variability to identify more targeted intervention at regional and global scale^8,24^.

This work is mainly relevant for policy makers looking at the hemispheric transport of air pollution where the global and regional picture of the emissions is needed to model heavy pollution events not only affected by local sources but also by transported pollution. Local authorities focusing on local pollution events might complement the EDGAR data with local (city or province scale) emission inventories.

## Methods

This section describes (i) how higher temporally distributed emissions (total country and sectorial emissions as well as spatially distributed emissions) are derived from annual emissions, (ii) how yearly, weekly and daily profiles (in terms of weighting factors) are developed.

### General approach to distribute annual emissions to high time resolution data

The general approach to account for the temporal variation of emissions is to distribute the annual total to monthly, daily and hourly emissions using yearly (12 coefficients for monthly variation in a year), weekly (7 coefficients for daily variation in a week), and daily (24 coefficients for hourly variation in a day) profiles. Temporal disaggregation of emission data may increase the range of data uncertainty as precise estimate of monthly, daily, and hourly distributions of emissions is quite complex and heterogeneous, in particular when operating at the global scale^25^. However, most of these studies are limited in scope with regard to the coverage in emission sources, time frames, and geographical regions.

Till present, the EDGAR database provides annual and monthly sector- and country- specific emission time series and maps but with some limitations that we are overtaking with this study. The ‘Online-only Table 1’ provides an overview of sector specific yearly profiles previously used by EDGAR^16^ and the current work (EDGAR_temporal_profiles_r1^20^). In Janssens-Maenhout *et al*.^16^ the distribution of yearly emissions to monthly data is mainly based on regional seasonal factors obtained from scientific literature (see ‘Online-only Table 1’) which are applied to all world countries based on their regional belonging (the Northern and Southern Hemispheres and the Equator). For the Southern Hemisphere, the Northern Hemisphere profiles were assumed shifted by six months, and for the countries in the equatorial region no seasonality was included (scaling factors of 1).

In this study, the most appropriate temporal profile for each EDGAR process is identified through a quality assessment procedure. All EDGAR processes (e.g. energy, industry, residential, transport, agriculture, etc., for a total number of 227 processes) and all countries (226 countries over the globe) are covered and allow the disaggregation of annual emissions over time for all co-emitted air pollutants and greenhouse gases by the same sources. The temporal profiles developed in this work can be applied to any greenhouse gas or air pollutant primarily co-emitted in the atmosphere by any IPCC emission reporting category (with the exception of Land Use, Land Use Change and Forestry (LULUCF) which is currently not included in EDGAR and in the temporal profiles). Particulate matter emissions from e.g. fireworks, wind erosion in agriculture, resuspension, etc. are not included in the EDGAR emission estimates and their seasonal pattern might need to be described through atmospheric models.

Combining annual emissions and temporal profiles, monthly and hourly disaggregated emission data for different sources and countries are generated for a representative year 2005 when no information over historic time series is available. The 2005 year is selected for this purpose because it is a relatively recent year without anomalies (e.g. in terms of climate, economy, etc.) and is a base year for many mitigation measures (e.g. Nationally Determined Contributions under the Paris Agreement).

Yearly, weekly, and daily profiles are integrated into the EDGAR database in order to disaggregate annual emissions into finer data; to distribute the annual emissions to hourly emissions per grid the following relationship is used:

where:

- E = Emissions;
- x = Country, sector, year and month specific activity;
- y = Country, sector and day specific activity;
- z = Country, sector, day, hour and time zone specific activity;
- n = month- and year- specific number of days;
- i = grid code (lon/lat);
- s = Sector;
- h = hour (from 1 to 24);
- c = Country;
- j = Year;
- m = Month (from 1 to 12);
- d = Weekday;
- t = Time zone

### Computing monthly emissions: region and country mapping

In order to distribute yearly emissions to monthly data, all world countries are grouped into 23 regions for which region-specific yearly profiles are defined. Regional yearly profiles are defined mainly based on three parameters: i) climate zones, ii) heating degree days (HDD), and iii) ecological zones, defined as following:

1. Seasonal cycles are different for the different climate zones (e.g. equator (band between ±30°N), Northern (above 30°N) and Southern Hemispheres (below 30°S)), consequently affecting the seasonal variation of the activities and emissions.
2. Weather conditions strongly affect the energy consumption and emissions, especially extremes of temperatures. HDD is the cumulative number of degrees by which the mean daily temperature falls below a given temperature called the “reference temperature” (usually 18 °C or 19 °C which is adequate for human comfort). A “degree day” is calculated as the difference between the reference temperature and the average of the maximum and minimum temperature over the day. HDD is regarded as a reliable indicator for appropriately accounting for the effect of weather on energy demand. Based on a review of HDD data sources, HDD data are collected from the CMCC-KAPSARC degree days database^26^, which provides average HDD over the last decades for 147 countries. For countries which are not included in the CMCC-KAPSARC database, extrapolation is made considering mainly geographical proximity.
3. Temporal variation of activities and emissions can also be different among various ecological zones, especially for agriculture and biomass burning. The Food and Agriculture Organization of the United Nations (FAO) defines ecological zones considering climatic variables such as mean 24-hour temperature, diurnal temperature range, sunshine fraction, wind speed, relative humidity, wet day frequency and precipitation^27^.

**Fig. 1:**
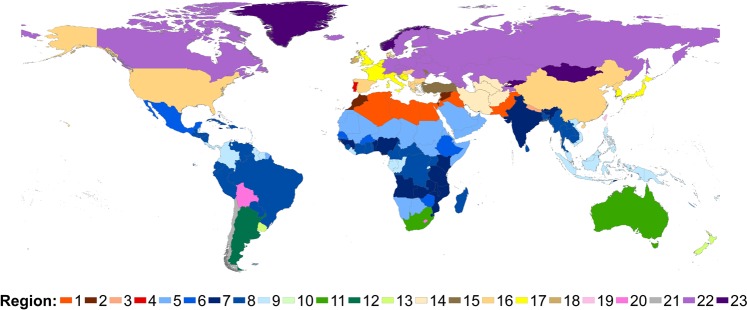
Regional aggregation of world countries for yearly profiles mapping.

Based on the above three parameters, all countries are grouped into 23 regions for yearly profiles mapping, as shown in Fig. 1. The definition of the regions is also listed in the file EDGAR_temporal_profiles_r1.xls.

### Computing weekly, daily and hourly emissions

The final stage of the methodology to disaggregate emissions in time is the production of hourly emissions at the global scale in form of total and/or sector specific emissions and gridded data. Weekend days, holidays, and time zone offset can be different for different countries and have relatively big influence on weekly and daily profiles. Therefore, to integrate weekly (daily share) and daily profiles (hourly share), the information on the day type (as defined in Table 1) of each day should be considered, since the hourly variation of the activities for certain sectors (e.g. transport) can be different on a weekday and on a holiday in different countries.

**Table 1:** Definition of day type.

Day type	Definition
1	Weekday
2	Weekend day one
3	Weekend day two or public holiday

The definition of the weekend days is also different among countries. Weekend types with different weekend days are defined for all world countries and mapped into EDGAR. Globally, there are six weekend types which are included in the EDGAR model, as specified by Table 2. Fixed and variable holidays are also compiled for all the countries over the 1970-nowadays time series. Coupling all these information with country- specific weekly and daily profiles into EDGAR, hourly emission time series and grid maps are produced.

**Table 2:** Definition of weekend type.

Weekend type	Weekend days
1	Friday
2	Friday, Saturday
3	Friday, Saturday, Sunday
4	Saturday, Sunday
5	Sunday
6	Thursday, Friday

In addition, to integrate the time zone information into EDGAR, time zone boundaries are extracted from time zone boundary builder (https://timezonedb.com/download). Country code, offsets from UTC, and summertime shift (from 1970 till nowadays, https://github.com/evansiroky/timezone-boundary-builder, https://www.timeanddate.com/time/dst/2005.html) are identified and compiled for all time zone regions. The development of such a system allows the representation of the hourly emissions during a specific day or heavy pollution episode in one global map.

### Development of temporal profiles for anthropogenic emissions

The basis of our work is the IER (Institute of Energy Economics and Rational Energy Use) database of temporal profiles to distribute the annual emissions from EDGAR, since it includes a large number of source- and country- specific temporal profiles developed within different studies^28–33^ and applied in several projects^34–38^. A review of temporal profiles from other studies and used by atmospheric models was also conduced. There are relatively limited amount of studies in this field. Studies have more focus on the monthly variations of emissions^4,11,12^, while little attention has been paid to daily and hourly variations^39,40^. The spatial and sectoral resolutions of relevant studies are also limited, often only for a specific region or sector^8,13,41^. Comparison of the IER database with the temporal profiles used by certain atmospheric models and other emission inventories shows good agreement across sectors, and a higher sector and region resolution in the IER database^42^.

The approach of deriving temporal profiles is based on statistical data sets (e.g. Eurostat, ENTSO-E, UN monthly bulletin, etc.) that can be used as proxy data for the temporal profile computation. The IER temporal profiles database covers the following main sectors: energy industry, fuels transformation/non-energy use, combustion in manufacturing industry, non-metallic mineral processes, chemical processes, metal processes, international and domestic aviation (distinguishing between cruise, climb and descent, take-off and landing), road transportation, non-road ground transport, international and domestic shipping; residential combustion, oil production and refineries, solvent use, agriculture, solid waste disposal, fossil fuel fires, large scale biomass burning.

Table 3 provides an overview of the indicator data used to represent the drivers of the temporal variations of activities and emissions of significant sources in the IER database. For example, fuel use and temperature are the main indicators for monthly variations of activities in power plants, industrial and small combustion plants. Daily and hourly variations of industrial activities are indicated by working times, time shifts and holidays. Temporal variations of transport activities are represented by traffic counts data.

**Table 3:** Driving forces (indicator data) for the temporal variations of activities and emissions of significant sources in the IER database^36^.

Sector	Indicator data for monthly variation	Indicator data for daily variation	Indicator data for hourly variation
Power plants	Fuel use Temperature	Load curves	Load curves
Industrial combustion plants	Production rate Fuel use Temperature	Working times Holidays	Working times
Small combustion plants	Fuel use Temperature	User behavior	User behavior
Refineries	Fuel use Oil throughput	Working times Holidays	Working times Shift times
Industrial processes	Production rate	Working times Holidays	Working times Shift times
Road transport	Traffic counts	Traffic counts	Hourly traffic

In addition to the information provided by the IER database, for the power generation, residential combustion and agriculture sectors, further developments of the yearly profiles have been included in the current work, as discussed in the next paragraphs. As major improvement compared to the IER database is the generation of time dependent temporal profiles for these sectors, reflecting the inter-annual variability of the key indicators used to temporarily distribute the annual emissions into monthly data. The ‘Online-only Table 1’ compares the data sources used to derive the yearly profiles in EDGAR_temporal_profiles_r1 and in the former version of the EDGAR database (EDGARv4.3.2^16^).

Due to the limited data availability for all countries, it is not always possible to consider regional or local characteristics for each country. Country-specific temporal profiles are extrapolated to the globe using information on climate zone, seasonal variation, average temperature, and other socio-economic parameters. Yearly, weekly, and daily profiles expressing the temporal variation of activities and emissions are then compiled in the database for all countries.

Concerning the hourly profiles development, the aim of this study is to build a system within the EDGAR database allowing the downscale of annual emissions to hourly data at gridcell level. The hourly profiles implemented in the current work do not always represent the best profile for each sector and country combination. However, having rather generic profiles reduces the discontinuity from cell to cell due to the application of the same hourly profile through all the countries. The end-users of the EDGAR data can however provide ad hoc temporal profiles to be implemented in the EDGAR system. Moreover, EDGAR data are often used in global chemical transport models which do not always require hourly emission data as input.

### Temporal profiles for power generation

Electricity production and fuel consumption in the power sector coupled with ambient temperature are considered as the indicators of the yearly variation of power generation emissions. In order to develop monthly profiles to be applied to the EDGAR power generation emissions, monthly electricity statistics are gathered from IEA (https://www.iea.org/statistics/monthly/#electricity) and other national statistics. IEA provides monthly statistics for electricity production (in GWh/month) for different type of fuels (natural gas, oil, coal, biofuels, other) for all OECD countries from January 2016 till 2018, while from January 2000 onwards the monthly statistics of produced electricity by country are not provided by fuel category. OECD countries include: Australia, Austria, Belgium, Canada, Chile, Czech Republic, Denmark, Estonia, Finland, France, Germany, Greece, Hungary, Iceland, Ireland, Italy, Japan, Korea, Latvia, Lithuania, Luxembourg, Mexico, Netherlands, New Zealand, Norway, Poland, Portugal, Slovak Republic, Slovenia, Spain, Sweden, Switzerland, Turkey, United Kingdom, United States. Therefore the same monthly profile is assumed for all fuels over those years. For the years before 2000, the same monthly profile as in 2000 is assumed. For China, information on the monthly GWh produced for all fuels is obtained from the National Bureau of Statistics of China (http://www.stats.gov.cn/english/), covering the 1990–2017 period. Regional averages are also computed using the country specific yearly profiles and applied to the remaining world countries consistently with the regional aggregation shown in Fig. 1. Emissions from the different power plant types included in the EDGAR database (i.e. auto producers electricity plants, auto producers heat plants, auto produced cogeneration, public cogeneration, public district heating, public electricity production, own use of electricity and heat) are distributed assuming the same temporal pattern of the electricity production, being the latter the major contributor of the emissions of the whole energy sector (e.g. in 2018, 76% of global fossil CO_2_ emissions from power generation are produced by public electricity generation). For completeness, profiles for nuclear power plants and pumped storage of electricity are estimated under the assumption of continuum operation (although they have a yearly shutdown for at least 3 weeks).

**Fig. 2:**
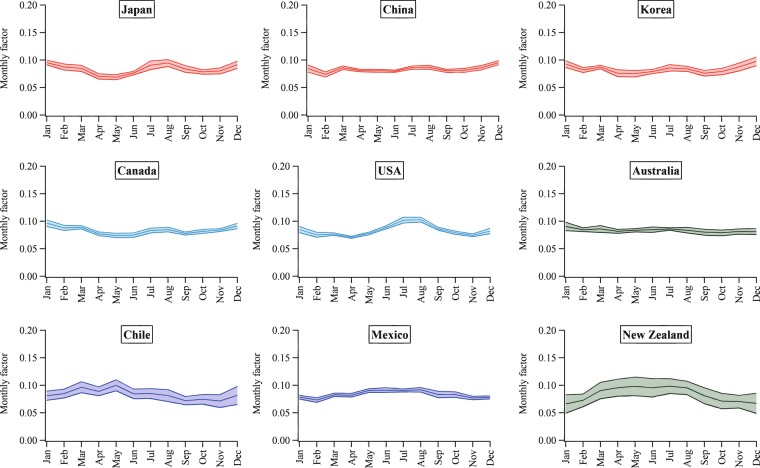
Inter-annual variability of monthly scaling factors over the 2000–2017 time series for the power generation sector for Asian countries (red), North America (light blue), Oceania (dark green) and Latin America (blue). Mean values ± one standard deviation are represented.

**Fig. 3:**
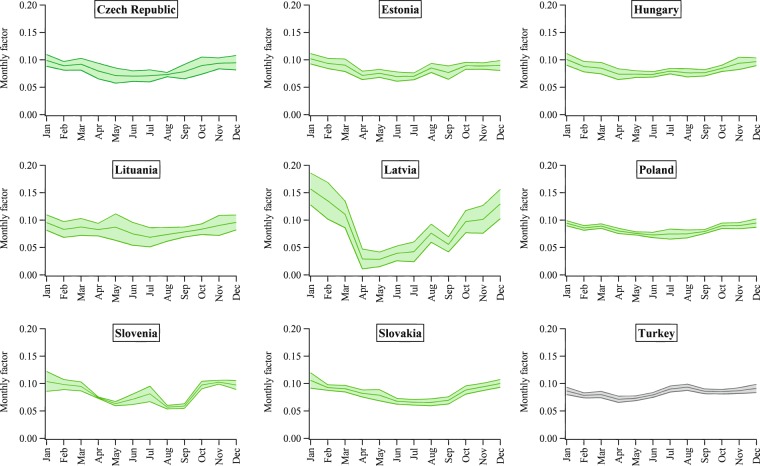
Inter-annual variability of monthly scaling factors over the 2000–2017 time series for the power generation sector for Central European countries (light green) and Turkey (grey). Mean values ± one standard deviation are represented.

**Fig. 4:**
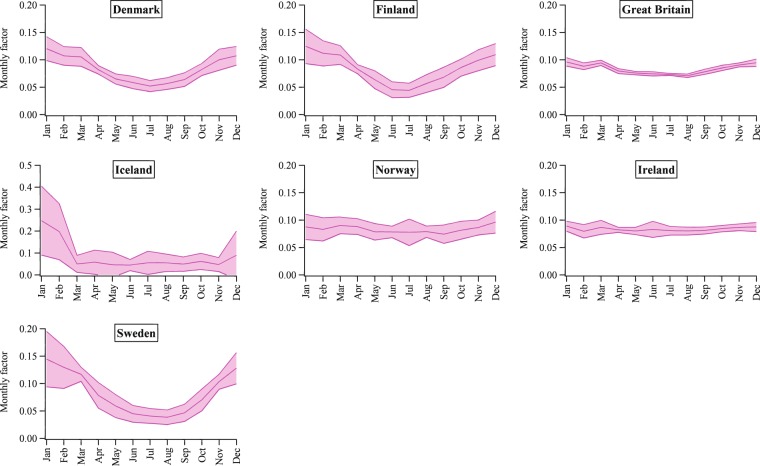
Inter-annual variability of monthly scaling factors over the 2000–2017 time series for the power generation sector for Northern European countries (pink). Mean values ± one standard deviation are represented.

**Fig. 5:**
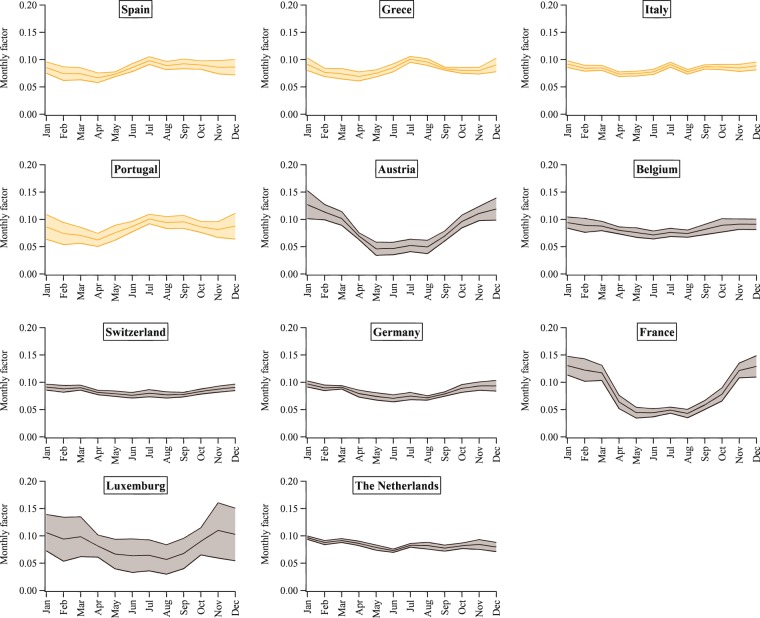
Inter-annual variability of monthly scaling factors over the 2000–2017 time series for the power generation sector for Southern European countries (yellow) and Western European countries (grey). Mean values ± one standard deviation are represented.

Figures 2–5 show the mean seasonal variations of the yearly profiles (i.e., monthly scale factors) obtained from the IEA monthly electricity statistics over the time period 2000–2017 for the 35 OECD countries and obtained from the National Bureau of Statistics of China over the time 1990–2017. This analysis allows us to evaluate the representativeness and stability of each profile for a given country over the years and to identify specific features of the monthly variation of the energy production depending on the geographical location of each country. Larger standard deviations occur for Lithuania, Latvia, Finland, Iceland, Norway and Sweden, meaning that for these countries larger variations in the power generation happen from one year to another probably due to changes in economic variables, meteorological conditions as well as electricity trade. The EDGAR database is not an input-output model and currently it does not include trade information which might have some effects also on the electricity patterns described in this work. In addition, data availability might influence the different standard deviations represented in Figs. 2–5. Higher contributions of the energy sector are expected over the cold months, while lower contributions are found over the warmer months (see as an example the seasonal pattern of Northern European countries represented in Fig. 4). A particular feature of the Southern European countries characterized by very hot summer periods is the presence of local peaks also over the summer months due to the energy production for cooling (see Fig. 5).

To calculate the daily variation, the influence of weekdays is taken into account, while to resolve hourly emissions typical national heat and electricity demand profiles are implemented accordingly with the methodology developed by Adolph^29^, showing lower contributions at night and characteristic peaks during the day time.

### Temporal profiles small scale combustion

Emissions from small scale combustion (e.g. the residential sector) show a significant seasonal variability, reflecting the climatic conditions of each country. In order to account for the ambient temperature dependence of the heating system in the residential sector, heating degree days (HDD) have been computed using the 2 meters temperature data with hourly resolution obtained from the ERA5 atmospheric reanalysis of global climate produced by ECMWF within the Copernicus frame over the years 1980 till 2017 (https://cds.climate.copernicus.eu/cdsapp#!/dataset/reanalysis-era5-single-levels?tab=overview). Hourly averages are computed using the temperature data available for each gridcell at 0.25° × 0.25° within each country, thus allowing the computing of country specific yearly profiles for this sector based on HDD (assuming a reference temperature of 18 °C for all countries to allow comparability amongst them). In addition to the seasonal variability associated with the HDD metric, a proportion of energy consumption is assumed for each of the 23 regions to be constantly used for the production of hot water in the residential sector based on the IER dataset^42^. EDGAR emissions from the residential sector are related with the combustion of fuels for heating and cooking in household/commercial/other purposes. Therefore, HDD is an indicator that suites this purposes globally (northern and southern hemispheres) and this is also the reason why the temporal profiles do not incorporate Cooling Degree Days (CDD) since mainly related with electricity consumption.”

To calculate hourly emissions for the residential sector, hourly patterns of fuel use for heating purposes and hourly patterns of production related fuel use following the typical daily working times are considered within the IER database. However, hourly variation of heating related fuel use depends on heating technology, insulation standards and climatic conditions, variables that are partly included in the current work. For central-heating, we assume a reduction of residential emissions at high ambient temperature, showing a reduction of residential emissions at night time. For coal or wood stoves, two daily peaks are observed, corresponding to the morning and late-afternoon or evening fueling of the stoves in the early morning and after returning home from work. In the IER database^42^, hourly patterns for households are derived from an evaluation of a comprehensive survey in Germany in VDI (Verein Deutscher Ingenieure) guideline 2067^43^. For commercial and institutional heat plants a similar hourly pattern of the household combustion activities is assumed due to the lack of information. Additional information from surveys in different small consumers groups conducted by Seier^32^ is also included.

### Temporal profiles for agriculture

Monthly variation of agricultural emissions can be quite different among different sub-sectors (e.g. animal emissions, manure emissions) and pollutants (e.g. CO_2_, CH_4_, NH_3_). Moreover, temporal variations of agricultural emissions can vary from one year to another as they are related to meteorological conditions, e.g. temperature, wind speed (Friedrich and Reis, 2004). Owing to the lack of data and studies and the complexity of the problem, yearly profiles for agriculture sector are associated with relatively higher uncertainties^36^. Atmospheric modelers often use as reference for the seasonality, the monthly CH_4_ emissions of rice^44^ (https://data.giss.nasa.gov/ch4_fung/). Among all cultivation activities, we dedicated special effort in including seasonal profiles for the rice cultivation sector, representing a relevant source of CH_4_ emissions in the world (ca 11% in 2012^16^). Emission from rice cultivation in top methane emitters countries like China and India (∼20% and ∼10% of global share) accounts for over 4% and 1% of total methane emission, respectively. For the other crop related emissions (wheat, maize, etc.), the assumption of constant production over the year is made, leaving developments for this subsectors to a future release.

The recent RiceAtlas^45^ produced by the International Rice Research Institute (IRRI) provides a comprehensive rice calendar with monthly specification at country to sub-country level, covering the whole world with monthly cultivated area and production data. The split between the different agro-ecological ecosystem types is already taken into account by EDGAR, in that four specific emission factors are implemented depending on the fraction of cultivated area: rainfed, deep water, irrigated, and upland (never flooded for a significant period of time).

**Fig. 6:**
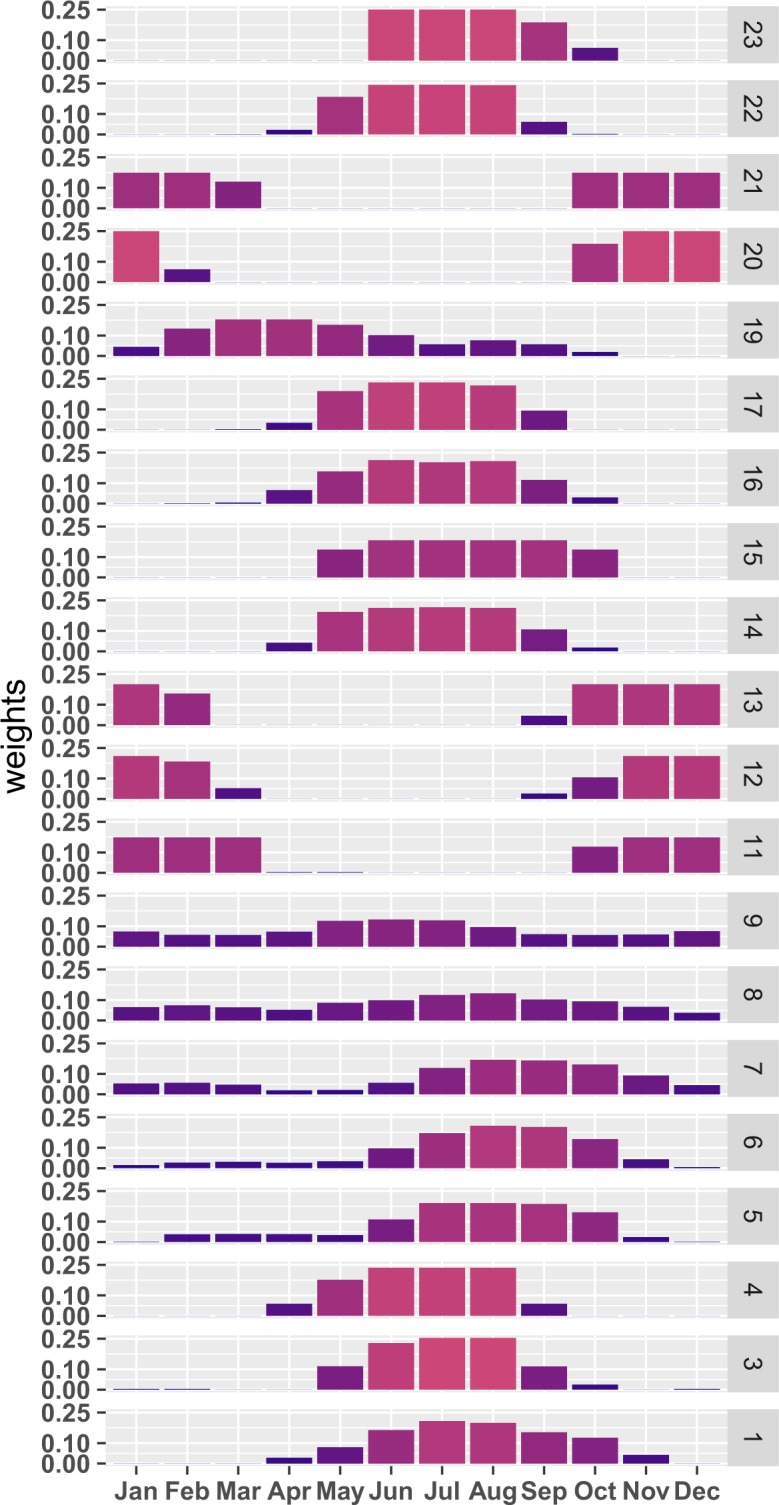
Monthly weights for rice-area and for the 23 world regions.

The month-varying weights in Fig. 6 represent the share of the area cultivated to rice as average in the years 2010–2012, as provided by the RiceAtlas, and aggregated for the sub-regions of Fig. 6. The seasonality reflects the multiple crop patterns during the majority of the year (e.g. regions 7, 8 and 9, including India and Indonesia), or concentrated during half year (typically from early/late spring to early/late autumn), as for example in region 16.

Laborte *et al*.^45^ explain the limitations of other existing rice calendars datasets, which include poor or outdates coverage, missing information about multiple crop per year. The RiceAtlas fits the purpose of providing EDGAR with the most updated and comprehensive monthly disaggregation available. Early studies by e.g. Matthews *et al*.^44^, based on FAO statistics for annual production and on a variety of sources to derive monthly splitting, provided an exhaustive compilation of monthly cultivated rice area and associated latitudinal methane emission, which has been used as reference for emission calculation by a number of studies (e.g. Bergamaschi *et al*.^46^). Comparing the time series of monthly rice calendar predicted by Matthews *et al*.^44^ and by the RiceAtlas for four among the top ten world rice producers, the profiles for China and India do not show any significant offset although a weak deviation in magnitude is observed. For USA, RiceAtlas predicts an earlier cultivation and quicker ending respect to Matthews *et al*.^44^, peaking in April-May rather than from May to September and terminating in October rather than November. For Indonesia, the difference between the two monthly series is quite substantial, with seasonal offset and difference in the peaking period, possibly due to change of practice or difficulty in retrieving monthly splitting for this country. In this sense, the consistency of the recent RiceAtlas datasets can be thought as a reliable update for compilation of emission inventories.

Animal related emissions estimates do not show a yearly pattern, since intensive production activities in this sector are kept rather constant. However, some variations in the emissions over the course of the year might be found in countries where animals are grazing in summer months and kept in stables in winter periods (or rainfall periods) and where animal feed varies considerably throughout the seasons. In this work we rely on the assumption of intensive animal production with no seasonality for indoor husbandry (mainly cattle, pigs and poultry), although representing a simplification of country specific practices which a global database like EDGAR cannot modulate.

Hourly emission patterns follow animal activity, which is mainly influenced by times of feeding, drinking and resting and thus shows a clear day/night-rhythm. Typical courses of emissions are examined in the work of Comberg & Wolfermann^47^, Hahne *et al*.^48^, Hinz & Linke^49^, Kaiser^50^ and Mayer^51^.

The temporal variability of emissions from manure management is mainly dependent on temperature and grazing periods, although the amplitude of this seasonality is rather uncertain. No detailed information is available about the hourly pattern of manure management, therefore a day/night-rhythm determined by the influence of temperature is assumed within the IER database^36^.

To our knowledge, no global data is available regarding enteric fermentation in ruminants and manure management (only few local measurements could be found^52,53^ to better describe the seasonal variation of animal related emissions.

### Temporal profiles for road transport

Road transport emissions are characterized by very strong seasonal and hourly variations. Temporal profiles included in this work are entirely based on the IER database^36^, whose methodology and assumptions are briefly summarized below.

Monthly variations of road transport emissions are gathered from monthly national energy statistics available from EUROSTAT^54^ for Europe. Additional information is also included from traffic flow data (e.g. traffic counts) although appropriate traffic counts data are often not available. While different vehicle types show a similar temporal behavior, different road types (e.g. motorways, rural and urban roads) show different temporal patterns. Stronger seasonal variation is found for motorways than for rural and urban roads, and a stronger seasonal variation in rural areas than in urban areas. Hourly variations, instead, are quite similar for different road types and regions. Whereas evaporation emissions mainly depend on temperature, exhaust emissions are connected with the variation of traffic volume. Road transport emissions are characterized by a very strong hourly variation, with daytime peaks up to 7 times higher than the lowest emissions happening at night time.

### Temporal profiles for other mobile sources

Other mobile sources include air, ship and railway traffic, and off-road vehicles. Landing and take-off cycles (LTO cycles), passenger numbers and freight statistics available from airports or the International Air Traffic Association (IATA) are used to generate profiles for aircraft emissions in the EDGAR database. However, in the EDGAR_temporal_profiles_r1^20^, only one yearly profile is defined for the flight components included in the EDGAR emissions (landing and take-off, cruise and climb-out and descent) since no differentiation of the monthly variation of these components is present. Modelers interested in low- and high-altitude emissions can directly use the EDGAR aviation emission gridmaps disaggregated for the different flight components or apply the same yearly profile to disaggregate their own emission data of each flight phase. Hourly emissions from air transport are assumed to be distributed over the day without strong variations.

To distribute shipping emissions, the number of ships per hour, day, week, or months in harbours or on ship routes could be used. However in this work, an equal distribution over the year and over the day is assumed due to the lack of appropriate data.

For off-road vehicles (e.g. construction machinery, agricultural machinery) a similar temporal variation of production processes in small enterprises (night/day, working day/weekend) is assumed. A strong seasonal variation is taken into account for activities of agricultural machinery^36^.

### Temporal profiles for waste

The temporal distribution applied to the waste sector in this work is based on the IER database^36^. Emissions from the waste sector include landfills, incineration plants and sewage treatment plants which are assumed to be constant during the year and with no significant weekly and daily patterns. Also for landfills, the seasonal changes in ambient temperature do not affect waste temperature in layers deeper than two meters below surface.

### Temporal profiles for other sources

The temporal patterns (monthly and hourly) of all remaining emission sources (e.g. refineries, industrial processes, fuel extraction, transformation, etc.) are taken from the IER database^36^.

## Data Records

The EDGAR temporal profiles library (EDGAR_temporal_profiles_r1^20^) and the emission time series of EDGARv4.3.2 with the new monthly resolution are available for all pollutants (CO_2_, CH_4_, N_2_O, SO_2_, NOx, CO, NMVOC, NH_3_, PM_10_, PM_2.5_, BC, OC) as Microsoft Excel files and can be freely accessed via the EDGAR website at http://edgar.jrc.ec.europa.eu/overview.php?v=temp_profile (last update: March 2020). We have applied the EDGAR_temporal_profiles_r1 to the EDGARv4.3.2 emission data since the latest EDGARv5.0 version currently covers only the GHGs and to allow EDGAR users to compare the monthly emission time series of EDGARv4.3.2 obtained with the former EDGAR temporal profiles (available at https://edgar.jrc.ec.europa.eu/overview.php?v=432_AP and https://edgar.jrc.ec.europa.eu/overview.php?v=432_GHG) with the new ones.

Data are registered at figshare^20^ and available also at https://data.europa.eu/doi/10.2904/JRC_DATASET_EDGAR ^55^. Monthly temporal profiles are downloadable online for each country (226 countries plus international shipping and aviation) and source category (e.g. energy, industry, residential, transport, agriculture, etc., for a total number of 227 processes). The EDGAR_temporal_profiles_r1^20^ are reported in the EDGAR_temporal_profiles_r1.xls file including the following information: region/country, activity sector description, IPCC_1996_source_category, IPCC_2006_source_category, year, yearly temporal profiles. The EDGAR temporal profiles library will be further improved for specific sectors or countries when new data will become available. New versions will be periodically uploaded to the JRC EDGAR Repository.

## Technical Validation

All datasets used to compile the temporal profiles database EDGAR_temporal_profiles_r1^20^ for global anthropogenic emissions are entirely based on official international statistics (e.g. IEA, NBSC, EUROSTAT, etc.), reputable data sources (ECMWF ERA5 atmospheric reanalysis of global climate, etc.) and relevant scientific literature works, which guarantee the robustness and validity of the underlying data used in this research.

Temporal allocation of emissions is often based on expert opinions as well as on the use of surrogate data, therefore limiting the information about the accuracy and the quality of the data. It is challenging to quantify the impact on uncertainty of some of the data handling steps, e.g. expert judgement in allocation of temporal profiles to a particular EDGAR sector. The results from such approach would be greatly influenced by the assumptions incorporated into the analysis. It is therefore considered that a qualitative analysis is the most appropriate method for expressing uncertainties, by using “quality scores” (Table 4). The use of “quality scores” is an approach commonly used when uncertainties are particularly large or difficult to quantify (as used in other studies, e.g. Huang *et al*.^56^). In this case, quality scores are assigned to an EDGAR source group to represent the quality (accuracy/appropriateness and completeness) of the temporal profiles that are assigned to the EDGAR sector. A quality assessment of the generated datasets is performed, showing the reliability of the temporal disaggregation in particular for combustion related sectors (e.g. power plants, transportation, residential combustion, etc.), while anticipating the need for further development for some fuel transformation activities, some agriculture sub-sectors (e.g. manure management, enteric fermentation, and agricultural waste burning) and waste management.

**Table 4:** Quality scores used to describe the quality of the match between the temporal profile and the EDGAR process.

Quality score	Description	% CO_2_ emissions in 2005
1	Well matched.	33
2	Sector-specific, sub-sector not differentiated.	38
3	Catch all processes, a general profile that provides a best available match.	23
4	Best profile available, not considered to be a specific match.	6

The EDGAR emission process code consists of five 3-letter codes which identify the sector, sub-sector, fuel type, technology and end-of-pipe measures related to the emissions. The temporal profiles mapping is performed mainly at sector and sub-sector level (first 2 levels of EDGAR code), considering that technology and end-of-pipe measures have little impacts on the temporal variation of the activities, and on the other hand the lack of fuel type specific temporal profiles. Table 5 lists the aggregated sectors of the EDGAR database for which different types of temporal profiles are assigned, together with representative quality scores.

**Table 5:** List of EDGAR sector codes and representative quality score for yearly profiles.

EDGAR code	Description	Representative quality score
AGS	Agricultural soils	1
AWB	Agricultural waste burning	4
BMB	Large scale biomass burning	1
CHE	Production of chemicals	1
ENE	Energy industry	1
ENF	Enteric fermentation	4
FFF	Fossil fuel fires	1
FOO	Production of foods	2
IDE	Indirect emissions from non-agricultural NH3 and NOx	4
IND	Combustion in manufacturing industry	1
IRO	Production of iron and steel	1
MNM	Manure management	4
N2O	Indirect N2O emissions	1
NEU	Non energy use of fuels	1
NFE	Production of non-ferrous metals	1
NMM	Production of non-metallic minerals	1
PAP	Production of pulp and paper	2
PRO	Fuel production/transmission	1, 2, 3
PRU	Production and use of other products	3
RCO	Residential	1
REF	Oil refineries	1
SOL	Application of solvents	1
SWD	Solid waste disposal	3
TNR	Non-road transport	1
TRF	Transformation industry	1, 2, 3
TRO	Road transport	1
WWT	Waste water	4

In order to show how well a temporal profile qualitatively matches the corresponding EDGAR process, quality scores indicating the level of the matching quality are assigned (see Table 5). Four levels of mapping quality scores, from 1 to 4, are defined to give an indication on how well a matching is and on priorities for further improvement for the yearly profiles. Quality scores 1 and 2 are considered to be a relatively good match and representative of the EDGAR sector. Quality scores 3 and 4 represent fuzzy matches due to the lack of process-specific temporal profiles and are considered to be the priority areas for further development. A quality code of 1 is assumed for oil, 2 for gas and 3 for coal related fuels for the fuel production sector (PRO). A quality code of 1 is assumed for combustion activities in the transformation industry sector, 2 for transformation in gas to liquids plants, chemical heat for electricity production, fuel transformation in gas works and non-specified transformation activity and 3 for blast furnaces, electric boilers, blended natural gas, heat pumps, gasification plants for biogas, charcoal production plants, coke ovens, transformation in liquefaction/regasification plants in gas to liquids plants, in coal liquefaction plants, in patent fuel plants, distribution losses in transformation processes, petrochemical industry.

Table 4 also presents the percentages of global CO_2_ emissions in 2005 associated with each quality code. 33% of CO_2_ emissions in 2005 are attributable to sources for which temporal profiles with quality code 1 (well matched) are mapped. 38% of CO_2_ emissions are associated with temporal profiles that are considered to be sector specific without fully differentiation of sub-sectors (i.e. quality code 2). 29% of CO_2_ emissions are attributable to the sources to which a general temporal profile is assigned (quality code 3 and 4). Lower mapping qualities are found for some fuel transformation activities and some agriculture sub-sectors (e.g. manure management, enteric fermentation, and agricultural waste burning), owing mainly to the fact that temporal variation of activities and emissions from these sectors are quite diverse and to lack of knowledge. Further improvements are therefore needed to develop more representative temporal profiles for these sources.

To further address the quality of the produced data set per sector and region, the assigned temporal profiles are compared with the temporal profiles used by common atmospheric models as discussed in the following section.

### Results comparison

Temporal profiles are commonly required by CTMs to distribute annual emissions to monthly and hourly emissions. To conduct a comprehensive comparison between the temporal profiles assigned to the EDGAR database in this study with other existing data sets, temporal profiles used by the CHIMERE^57^ and LOTOS-EUROS^58^ models are reviewed and collected. The monthly profiles used in the EDGAR v4.3.2 database, the Hemispheric Transport of Air Pollution v2 (HTAP) Task Force^59^ and the Community Emissions Data System (CEDS)^60^ are also included in the comparison.

Table 6 summarizes the main characteristics of the six data sets considered in the comparison regarding spatial, sectorial and temporal resolution. CHIMERE and LOTOS-EUROS profiles cover the 28 European countries. HTAP temporal profiles were developed for Europe, the United States of America (USA), and Canada. Temporal profiles from EDGAR v4.3.2 are global; however, they only distinguish three geo-regions, i.e. the Northern temperate zone, equator, and the Southern temperate zone. This study (226 countries) and the CEDS (222 countries) inventory provide country-specific temporal profiles for global emission databases. However, the CEDS inventory, similar to HTAP and EDGAR v4.3.2, only has yearly profiles.

**Table 6:** Overview of the compared data sets.

Data set	Spatial resolution	Sectorial resolution	Temporal resolution
This study	Global, country-specific	20 sectors	yearly, monthly, weekly, daily, hourly
CHIMERE	28 European countries	11 sectors	yearly, monthly, weekly, daily, hourly
LOTOS-EUROS	28 European countries	11 sectors	yearly, monthly, weekly, daily, hourly
EDGARv4.3.2^16^	Global, 3 geo-regions	15 sectors	yearly, monthly
HTAP	USA, Canada, Europe, China	6 sectors	yearly, monthly
CEDS	Global, country-specific	7 sectors	yearly, monthly

The CHIMERE and LOTOS-EUROS temporal profiles apply Standardized Nomenclature for Air Pollutants (SNAP) sector categorization, and differentiate 11 SNAP sectors. The HTAP and CEDS data sets have a relatively rougher sector resolution (6 and 7 main sectors, respectively). The EDGAR v4.3.2 data set integrates temporal profiles for 15 main sectors. In this study, temporal profiles covering 20 main sectors are mapped to all EDGAR processes.

Regarding temporal resolution, the EDGAR v4.3.2, CEDS, and HTAP data sets have only yearly profiles with monthly variations. CHIMERE, LOTOS-EUROS and this study employ yearly, weekly and daily profiles, and therefore enable emission distribution not only to monthly scale but also to hourly values.

In addition, a very important novelty of our work is the development of year dependent monthly scaling factors for activities with strong inter-annual variability. This information is absent in the aforementioned literature datasets.

### Global monthly emission pattern

**Fig. 7:**
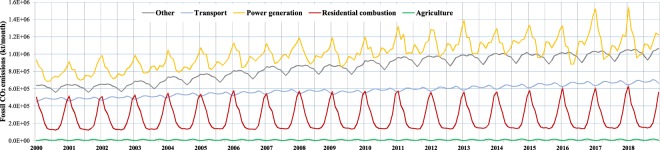
Time series (2000–2018) of monthly fossil CO_2_ emissions by sector in the world.

**Fig. 8:**
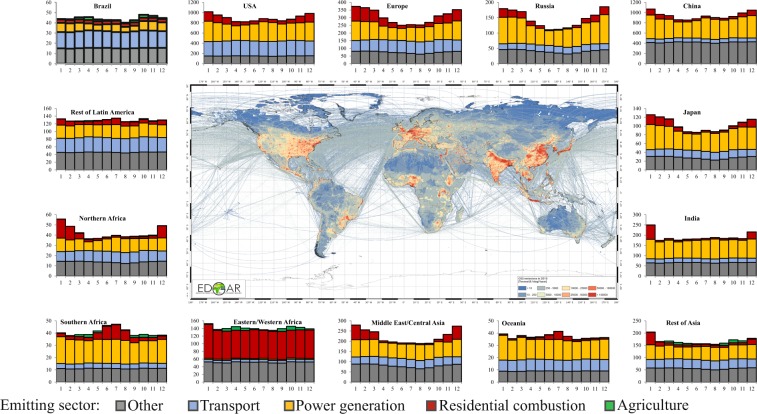
Seasonality of regional fossil CO_2_ emissions in 2015 (expressed in Mt/month).

**Fig. 9:**
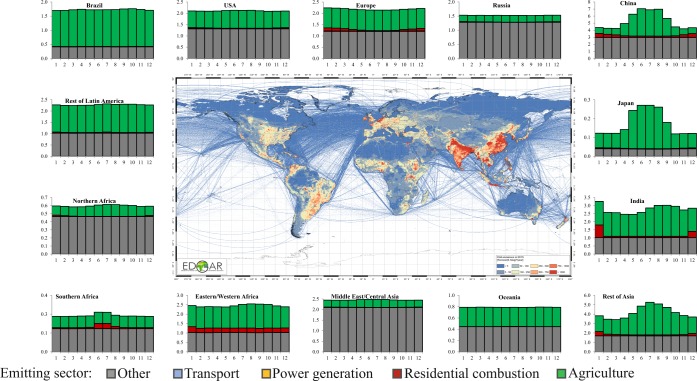
Seasonality of regional CH_4_ emissions in 2015 (expressed in Mt/month).

When applying the EDGAR_temporal_profiles_r1^20^ to the EDGARv5.0 emissions^61^, https://edgar.jrc.ec.europa.eu/overview.php?v=booklet2019), the corresponding monthly data successfully reproduce the major seasonal patterns that are expected, as shown in Figs. 7–9.

Figure 7 shows the time series of monthly fossil CO_2_ emissions from the year 2000 to 2018 for sectors with a strong temporal variability over the course of the year^61^. As top CO_2_ emitting countries are located in the Northern Hemisphere (e.g. China, USA, Europe, Russia), the monthly variations of global CO_2_ emissions are dominated by the seasonal variations of the Northern Hemisphere countries. Therefore the annual variability of global CO_2_ emission is strongly influenced by the power generation and residential combustion sectors, with higher emissions during the winter months and lower emissions from May to August. The residential sector is the one showing the strongest monthly variation, with emission peaks during cold months more than 3 times higher than the summer time peaks. Emissions from the agricultural sectors have an anti-correlated seasonal cycle compared with combustion related emissions due to the occurrence of higher agricultural emissions over the warmer months. This pattern is also enhanced when looking at the seasonality of CH_4_ emissions (Fig. 9), showing strong temporal variability in the agricultural sector anti-correlated with the residential combustion activities. As global average, agricultural activities are characterized by emission peaks during warmer months 1.5 times higher than during cold months.

Figure 8 represents the seasonality of fossil CO_2_ and Fig. 9 of CH_4_ emissions in 2015 by sector for different regions in the world. The highest contributions to CO_2_ emissions happen in the Northern Hemisphere during cold months mainly due to the combustion of fuels in the power and residential sectors. Specific seasonality is observed in Brazil and Latin America for the agricultural sector mainly from agricultural waste burning activities and agricultural soil emissions. The seasonality of CH_4_ emissions is mostly dominated by agricultural activities, in particular in countries with high emissions from rice cultivation which reflect the rice cultivation calendar (e.g. China, India, Japan, Rest of Asia).

## Usage Notes

The unique feature of the EDGAR_temporal_profiles_r1^20^ library relies on the possibility to use the newly developed sector- and country- specific temporal profiles i) as EDGAR application using data from in any EDGAR release, ii) by global and regional atmospheric modelers and iii) by emission inventory developers. The aim of this work is not only to improve the current knowledge of highly time resolved emissions, but in particular to allow any modeler to implement these new temporal profiles in any model, thus allowing the analysis of the impact of different emission temporal disaggregation methods on the model output. In addition, this work provides the basis for emission inventory developers aiming at disaggregating their annual emissions into higher resolution data.

In order to allow a straightforward implementation of the EDGAR_temporal_profiles_r1^20^ in any other system (emission database or model), each sector specific temporal profile has been mapped with a sector description and the standard Intergovernamental Panel on Climate Change (IPCC) 1996 (https://www.ipcc-nggip.iges.or.jp/public/gl/guidelin/ch1ri.pdf) and 2006 (https://www.ipcc-nggip.iges.or.jp/public/2006gl/pdf/1_Volume1/V1_4_Ch4_MethodChoice.pdf) classification and definition of source categories. Similarly, countries are identified with their name, regional belonging and International Organization for Standardization (ISO 3166-1 alpha-3 standard) codes in order to allow a clear and unique identification by any user.

Country names are consistent with the Interinstitutional Style Guide of the European Commission available at http://publications.europa.eu/code/en/en-370100.htm, the “Short name” definition listed in the “List of countries, territories and currencies” table at http://publications.europa.eu/code/en/en-5000500.htm has been used (updated at 16/07/2019).

## Data Availability

Most of the temporal profiles data processing has been done using the software R version 3.5 and Python version 3.6. The computation of heating degree days maps was based on the 2 m air temperature of ECMWF ERA5 re-analysis^62^ and produced by using IDL8.6 programming software. Further computations, such as mapping sectors and countries have been performed using Microsoft Access 2010. The implementation of the EDGAR_temporal_profiles_r1 library into the Emissions Database for Global Atmospheric Research has been developed using a dedicated EDGAR development tool of the Joint Research Centre named EOLO based on Php and Microsoft SQL Server. This system cannot be accessed outside the institution but further details can be provided upon request. All scripts related with this work are available at figshare^20^.

## Online-only Table

**Online-only Table 1:** Overview of sector specific yearly temporal profiles of the current work (EDGAR_temporal_profiles_r1) and the previous EDGAR profiles.

EDGAR code	EDGAR sector description	Previous EDGAR temporal profiles (for the Northern Hemisphere)	EDGAR_temporal_profiles_r1	
AGS	Agricultural soils	Based on agricultural model^63^	Based on the IRRI Rice Atlas^45^	country/region specific
AWB	Agricultural waste burning	Based on agricultural model^63^	Based on the IER database^36^	region specific
BMB	Large scale biomass burning	Constant profile	Constant profile	—
CHE	Production of chemicals	Constant profile	Based on the IER database^36^	region specific
ENE	Energy industry	Based on LOTOS^44^ and Builtjes^64^	IEA and NBSC monthly electricity statistics	year and country/region specific
ENF	Enteric fermentation	Based on agricultural model^63^	Constant profile	—
FFF	Fossil fuel fires	Constant profile	Constant profile	country/region specific
FOO	Production of foods	Constant profile	Constant profile	—
IDE	Indirect emissions	Constant profile	Constant profile	—
IND	Combustion in manufacturing industry	Based on LOTOS^44^ and Builtjes^64^	Based on Müller e*t al*.^35^ and Seier^32^	region specific
IRO	Production of iron and steel	Constant profile	Constant profile	—
MNM	Manure management	Based on agricultural model^63^	Constant profile	—
N2O	Indirect N2O emissions	Constant profile	Constant profile	—
NEU	Non energy use of fuels	Based on LOTOS^44^ and Builtjes^64^	Constant profile	—
NFE	Production of non-ferrous metals	Constant profile	Based on the IER database^36^	region specific
NMM	Production of non-metallic minerals	Constant profile	Based on the IER database^36^	region specific
PAP	Production of pulp and paper	Constant profile	Constant profile	—
PRO	Fuel production/transmission	Constant profile	Constant profile	—
PRU	Production and use of other products	Constant profile	Constant profile	—
RCO	Residential	Based on GENEMIS^34^	Based on HDD calculated from ERA5 atmospheric reanalysis of global climate produced by ECMWF^62^	year and country specific
REF	Oil refineries	Constant profile	Constant profile	—
SOL	Application of solvents	Based on Friedrich^65^	Based on the IER database^36^	country/region specific
SWD	Solid waste disposal	Constant profile	Constant profile	—
TNR	Non-road transport	Based on Wang et al.^66^ and Eyers *et al*.^67^	Based on the IER database^36^	country/region specific
TRF	Transformation industry	Constant profile	Constant profile	—
TRO	Road transport	Based on Friedrich^65^	Based on the IER database^36^	country/region specific
WWT	Waste water	Constant profile	Constant profile	—

[1]In Janssens-Maenhout *et al*. ^16^, the yearly profiles are defined for the Northern Hemisphere (without country specific definitions) and are shifted by 6 months to represent the yearly variability in the Southern Hemisphere. No seasonality is assumed for Equatorial regions.
